# Effect of Macro and Microalgae Addition on Nutritional, Physicochemical, Sensorial, and Functional Properties of a Vegetable Cream

**DOI:** 10.3390/foods13111651

**Published:** 2024-05-25

**Authors:** Teresa Muela, Adela Abellán, Cindy Bande-De León, Pablo Gómez, María Dolores Gil

**Affiliations:** Department of Nutrition and Food Technology, Universidad Católica de Murcia-UCAM, Campus de los Jerónimos, 30107 Murcia, Spain; aabellan@ucam.edu (A.A.); cmbande@ucam.edu (C.B.-D.L.); pgomez2@ucam.edu (P.G.); mdgil3@ucam.edu (M.D.G.)

**Keywords:** algae, *Chlorella vulgaris* golden, *Ascophyllum nodosum*, functional properties, sensorial profile, nutritional labeling

## Abstract

Algae are a booming resource in the food industry due to their several health benefits. This study assesses the impact of the combined use of selected macro- and microalgae to improve the nutritional profile and the labeling of a vegetable cream by the introduction of nutrition and health claims. As macroalgae, two *Ascophyllum nodosum* L., one natural (An) and one smoked (AnS), were selected for their high iodine concentration and flavor notes. A new strain of *Chlorella vulgaris*, golden (CvG), was selected as the microalgae, which is rich in proteins and has a neutral sensorial profile (golden color and mild flavor). In this study, two vegetable creams were compared. The control (CTRL) versus one enriched with a mixture of macroalgae and microalgae (CV-AN). Sensory, physicochemical, and functional properties of both vegetable creams were evaluated. The bioactivity assessed was the effect of iodine as a health claim and antioxidant and antihypertensive properties. CV-AN vegetable cream showed significantly higher values (*p* < 0.05) for protein content, iodine value, and antioxidant activity, with no significant differences (*p* > 0.05) in antihypertensive activity or sensory panel. The incorporation of these algae resulted in a vegetable cream with a better nutritional profile and sensory acceptability comparable to the control, offering protein and iodine source claims in the labeling.

## 1. Introduction

Algae have been part of Asian cuisines for centuries, valued for their rich concentrations of vitamins, minerals, proteins, fibers, and numerous other bioactive compounds. The biodiversity of compounds in algae can provide the potential for an innovative food industry focused on their production and use in functional products. The incorporation of algae in food could provide benefits for human health due to their bioactive compounds. Algae proteins have already been used in human and animal food, nutricosmetics, dietary supplements, among others [1]. 

Global consumption and production of algae have experienced significant growth in recent decades and are estimated at several million metric tons per year considering that these values may vary by region and consumption methods, as algae have multiple applications [2]. Some of the drawbacks of intensive algae cultivation are the possible negative environmental impacts, such as the alteration of marine ecosystems, competition with native species, and modification of the coastal landscape, as well as the limitations to remove these algae masses from the coasts [3]. 

In addition to their role in Asian cuisine, they have gained popularity worldwide, as they are also used in the production of food additives and supplements. China and Japan have the highest production and consumption of dried algae in the world. According to data from the Korean National Health and Nutrition Survey, algae consumption in Japan is 8.5 g/day although it can reach more than 10 g/day [2].

Currently, Japan is the world leader in the consumption of *Chlorella vulgaris* [4], and *Ascophyllum nodosum* is harvested and consumed in Europe at an annual rate of approximately 0.1 metric tons fresh weight per year, being one of the main species traditionally harvested in countries such as Ireland, Iceland, and Norway and, to a lesser extent, in France [5].

*C. vulgaris* is widespread in a wide range of regions around the world, as it has been recorded in various locations, including Namibia, India, Central America, Oceania, Antarctica, and the Arctic. Although its specific origin is not clearly defined, it has been identified in a variety of mainly freshwater aquatic habitats such as lakes, ponds, rivers, and streams. Prevalence and abundance may vary according to environmental factors such as nutrient availability and water temperature. This suggests a global distribution and adaptability to different conditions [6].

*C. vulgaris* is one of the most widely used species in food due to its use for several decades. The influence of this microalgae incorporation on the sensory acceptance of food products varies according to the composition of the food matrix. The acceptability and palatability of algae are negatively affected due to their inherent off-flavors, unusual dark colors, and strong odors. *C. vulgaris* is known for its intense vegetal taste and aroma, resembling powdered green tea. In addition, its intense green color could be considered a limitation in combination with a wide variety of foods [7]. Nevertheless, there are studies where the incorporation of *C. vulgaris* does not worsen the sensory acceptability of a variety of foods such as meat products [8], sausage [9] and hamburger [10], breadsticks [11], smoothies [12], and pasta [13]. However, to the authors’ knowledge, there are no studies focused on the use of the algal mixture proposed in this study for the reformulation of vegetable cream. The multiple and beneficial components of *C. vulgaris*, such as essential amino acids, antioxidants, minerals, omega-3 fatty acids, and dietary fibers, among others, are positively related to favorable results in the control and treatment of chronic diseases, such as vascular diseases, by improving lipid levels, inflammatory markers, oxidative stress, and blood pressure [14].

*Ascophyllum nodosum* is a brown alga commonly found in the North Atlantic, particularly along the coastlines of Europe and North America. It thrives in rocky environments and cold and temperate waters. It is a very adaptable species that can grow in a wide range of salinity and light conditions, playing a significant role in the coastal ecosystems it inhabits [15]. 

Fucoxanthin is the brown algae carotenoid. This pigment has demonstrated remarkable biological activities such as antioxidant, anti-diabetic, anti-inflammatory, anti-obesity, antimicrobial, cardiovascular protection, skin protection, neuroprotective, and cytotoxic effects against different cancer cell lines [16]. *A. nodosum*, being a large brown macroalgae, has a higher concentration of phenolic compounds than others, especially tocopherol and phlorotannin (tannins characteristic of brown algae), which results in a valuable source of antioxidants [17,18]. Polyphenols from *A. nodosum* algae are also known to have many health benefits due to their high antioxidant capacity, offering improvements against inflammation [19] and reduction in DNA damage in obese populations [20]. Moreover, *A. nodosum* promotes the ratio of Bacteroidetes to Firmicutes bacteria and substantially enhances the production of short-chain fatty acids, becoming a functional ingredient with the potential to improve intestinal health, which is closely related to obesity [21]. 

*A. nodosum* is widely used in the agricultural [22,23,24] and livestock [25,26,27] sectors, but some applications have been found in the food industry, suggesting the possibility of integrating them in gluten-free bread formulations in order to delay the aging of the product [28]. Other research conducted with bread showed that fortifying this product with A. *nodosum* had a significant effect on reducing energy intake at the next meal, suggesting that this algae could have the potential to affect body mass index or body composition positively [29].

The recommended daily intake of microalgae can vary depending on the specific type and intended use. While some microalgae have general dosage guidelines, it is essential to consider individual factors such as age, weight, overall health, and the intended purpose of consumption. For instance, *C. vulgaris* typically comes with a manufacturer’s recommendation doses per day. However, it is advisable to start with small doses and increase the dose according to the needs.

Regarding macroalgae consumption, the European Commission suggested that a moderate intake of macroalgae with high iodine content is advisable for adults. However, occasional consumption is recommended for young children and pregnant women to avoid the risk of excessive iodine intake, which could negatively affect thyroid function, especially during periods of growth and development. In addition, they can accumulate heavy metals and other pollutants if they grow in polluted waters. This could represent a health risk if they are consumed without adequate quality control [30].

Two organic products made from *A. nodosum*, of the Fucaceae family, harvested in the Scottish Outer Hebrides (Scotland, UK), have been selected as macroalgae: PureSea^®^ Natural (An) and PureSea^®^ Smoked (AnS) (Naturally Oak Smoked). Both have a high iodine concentration (650–980 µg/g) and provide umami and smoked flavor notes, respectively. Geographical location, growing conditions, and time of harvest are some of the factors affecting the amount of iodine contained in *Ascophyllum nodosum*. Generally, it can contain between 500 and 2100 micrograms of iodine per gram of dried algae [25,31]. As microalgae, *C. vulgaris*, of the Chlorellaceae family, was singled out, which is particularly interesting for its protein content, usually reaching 50–60% of the dry matter [32]. A strain of *C. vulgaris* with specific mutagenesis DNA, the golden *Chlorella vulgaris* (CvG), was selected. This product is commercialized under the trademark Alver Golden Chlorella^®^, and in addition to being rich in protein, 47.9 g/100 g according to the manufacturer, it has a neutral sensorial profile (golden color and mild flavor) and interesting technological properties. 

An increasing number of foodstuffs include nutrition and health claims in their labeling, packaging, and marketing. European legislation focuses on ensuring consumer protection and promoting healthy choices. This includes regulations to ensure the accuracy, transparency, and reliability of nutrition and health claims on foods. Optimization of the nutritional profile of foods fortified with micro- and macroalgae makes it possible to improve labeling by incorporating nutrition and health claims [33,34].

The aim of this study was to evaluate the impact of the combined use of specifically selected macro- and microalgae to improve the formulation of a vegetable cream inspired by a Spanish recipe comparing the nutritional, functional, and sensory properties of the control vegetable cream versus an algae–vegetable cream enriched with macro- and microalgae.

## 2. Materials and Methods

### 2.1. Formulation and Processing of the Algae–Vegetable Cream

The fermented and dried microalgae golden *Chlorella vulgaris* (CvG) under the trademark Alver Golden Chlorella ^®^ was supplied by the company Alver (Saint-Aubin, Switzerland). The dried and milled macroalgae *Ascophyllum nodosum* (An) and smoked *Ascophyllum nodosum* (AnS) under the trademark PureSea^®^ were purchased from Seaweed & Co (Whitley Bay, UK). 

The rest of ingredients to make the vegetable cream, frozen leek and carrot, Extra Virgin Olive Oil (EVOO), dehydrated potato flakes, and salt, were purchased from MAKRO market (Murcia, Spain).

Vegetable cream was made according to the following recipe: 30.5% frozen leek (*w*/*v*), 8.7% frozen carrot (*w*/*v*), 4.9% EVOO (*w*/*v*), 5.5% dehydrated mashed potato flakes (*w*/*v*), 0.6% salt (*w*/*v*), and 49.8% mineral water (*v*/*v*). This process was accomplished at the gastronomy laboratory “Gastrolab” of the Universidad Católica San Antonio (Murcia, Spain).

Two samples of 1.5 kg were made: control sample (CTRL) and enriched sample (CV-AN) with CvG at 3.3% (*w*/*w*) and An plus AnS at 0.2% (*w*/*w*). Seaweed concentrations were previously studied to formulate vegetable cream as a source of protein and iodine, under the standards for nutrition and health claims regulated by the European Food Safety Authority (EFSA) [33,34]. According to the technical specifications provided by the respective suppliers, CvG has an average protein content of 47.9%, while An and AnS have an average iodine concentration of 650–980 µg/g. Knowing these data and the nutritional composition of each ingredient, a theoretical nutritional composition of the vegetable cream was made from which the percentage of each algae to be added in order to label the cream as a source of protein and iodine was calculated.

In order to prepare the vegetable cream, leek, carrot, EVOO, salt, and mineral water were boiled for 20 min. The dehydrated potato flakes were added and cooked for an additional 4 min. The mixture was ground with a blender (Robot Coupé, Mini MP 190V.V.) at maximum speed for 2 min and passed through a sieve with a mesh size of 2 mm diameter. 

In CV-AN enriched sample, the mixture of macro- and microalgae was added after the end of the cooking stage and homogenized with the rest of the product during the grinding stage.

The samples were packed in 150 g vacuum bags (METRO Vacuum cooking bags, Smooth 80 µm OPA/PP) and thermally pasteurized (75 °C, 15 s) using an industrial convection oven (RATIONAL, SelfCooking Centre ^®^, Mod.SCC 61 WE). Afterwards, the samples were placed in the blast chiller (Abbattitore 5T, E-ABB5T) until their internal temperature reached 8 °C.

### 2.2. Physicochemical and Nutritional Properties

#### 2.2.1. Physicochemical Composition

Water activity (a_w_) was measured in 5 g of sample by an HP23-a_w_ Water Activity Meter (Rotronic, Bassersdorf, Switzerland), and pH was measured introducing the probe directly into the sample with the ph-meter Sension+ PH3, Hach (Loveland, CO, USA). Salt (NaCl) content was measured with an LAQUAtwin Salt-22 kit (Horiba Advanced Techno Co., Kyoto, Japan) in a water solution of the sample 1:10 (*w*/*v*).

To analyze the ash, Method 935.42 of the A.O.A.C. (2002) [35] was used. The porcelain crucibles were previously dried in the oven at 105 °C and weighed. The sample, previously dried for 24 h in an oven at 105 °C, was introduced into the crucibles and incinerated in a muffle furnace Select-Horn-TFT (JP. Selecta, Barcelona, Spain) at 525 °C for 24 h. After this time, the samples were cooled to room temperature in a desiccator and weighed. The measurement was made by difference in weights. To measure moisture, an automatic moisture analyzer MA 110.R (Radwag, Poland, EU) was used, in which 5 g of sample was dried at 105 °C. Fat analysis was performed according to ISO 14156:2001 [36] where a diethyl ether organic solvent was used as a solvent with an ST 255 Soxtec™ (FOSS, Denmark). Protein content was determined by the Kjeldahl method according to ISO 8968-1:2014 [37] using a digestion unit, Büchi SpeedDigester Unit K-436 (Büchi Labortechnik AG, Flawil, Switzerland). Then, a protein distillation unit, Büchi MultiKjel K-365 (Büchi Labortechnik AG, Flawil, Switzerland), was used and distilled with 70 mL of 40% NaOH and 10 mL of distilled water. The distillation content was poured into an Erlenmeyer flask with 25 mL of 4% boric acid with methyl red indicator. After distillation, titration was carried out with 0.1 N HCl, obtaining the total nitrogen (TN) value, which was multiplied by the conversion factor 6.25 to obtain the crude protein value. Amino acid nitrogen (AAN) and non-protein nitrogen (NPN) fractions were determined following the procedures described by Tejada, Abellán, Cayuela, et al. (2008) [38].

Fiber content was determined by FT 121 and FT 122 Fibertec™, FOSS (Hilleroed, Denmark) according to the standard EN ISO 13906:2008 [39].

All these measurements were conducted in triplicate.

#### 2.2.2. Iodine Content

Determination of iodine by ICP-MS (inductively coupled plasma mass spectrometry) was analyzed according to UNE-EN 15111:2007 [40] method in accredited laboratory, Eurofins Ecosur S. A. (Murcia, Spain). ICP-MS is an analytical method known for its high sensitivity to detect and quantify elements present at trace levels [41]. Measurements were made in triplicate.

#### 2.2.3. Colorimetry

Triplicates of each sample were uniformly distributed in Petri dishes (Thermo Fisher Scientific™, Madrid, Spain), and color parameters were measured with a Konica Minolta CR-410 Chroma Meter (Minolta, Osaka, Japan). The results were presented following the CIELAB system, where lightness was shown as L* (L* = 0 indicates black; the higher the lightness), and the chromaticity coordinates included the following: a* indicates redness degree (−a*: green, +a*: red), and b* indicates yellowness degree (−b*: blue, +b*: yellow). 

To show the color difference between the two samples, the Delta E (ΔE*) was used through the equation

(1) $$\Delta E*=\sqrt{({L*}_{\mathrm{CV}\text{-}\mathrm{AN}}-{L*}_{\mathrm{CTRL}}{)}^{2}+({a*}_{\mathrm{CV}\text{-}\mathrm{AN}}-{a*}_{\mathrm{CTRL}}{)}^{2}+({b*}_{\mathrm{CV}\text{-}\mathrm{AN}}-{b*}_{\mathrm{CTRL}}{)}^{2}}$$

where L* _CV-AN_, a* _CV-AN_, and b* _CV-AN_ are color parameters of algae–vegetable cream, and L* _CTRL_, a* _CTRL_, and b* _CTRL_ are the control vegetable cream color parameters.

### 2.3. Assessment of Bioactivity

#### 2.3.1. Antihypertensive Activity

Angiotensin I-converting enzyme (ACE-I) inhibitory activity was determined according to the method of Cushman and Cheung (1971) [42], modified by Miguel et al. (2004) [43]. ACE-I acts on the substrate used in the method, hippuryl-L-histidyl-L-L-leucine (HHL), releasing hippuric acid. The method is based on the spectrophotometric measurement at 228 nm of the absorbance of the hippuric acid released in the reaction. 

To assess antihypertensive capacity, 40 µL of non-protein nitrogen fraction of the sample was incubated at 37 °C with 100 µL of 5 mM HHL dissolved in 0.1 M borate buffer and 0.3 M NaCl (pH 8.3). Subsequently, 2 mU of ECA was introduced into the substrate, and after thirty minutes, 150 µL of 1 M HCl was added. The resulting hippuric acid was extracted with 1000 µL of ethyl acetate (which was subsequently removed by heating at 95 °C) and centrifuged at 4000× *g* for 10 min, after which the organic phase (800 µL) was collected. The resulting hippuric acid was reconstituted in 1000 µL of distilled water, and the absorbance was measured at 228 nm. 

To determine the concentration of peptide required to inhibit 50% of ACE activity, dilutions were prepared with varying concentrations of the samples, and the IC_50_ was calculated. A graph was then constructed plotting the percentage of ACE-I activity versus the concentration of sample used (µg peptide/mL). 

To determine the sample’s ACE-I inhibitory activity, the IC_50_ was calculated. Results were expressed in µg/mL ± standard error of the mean. The measurements were conducted in triplicate.

#### 2.3.2. Antioxidant Capacity 

The determination of antioxidant activity was performed using the 2,2-diphenyl-1-picrylhydrazyl radical (DPPH), following the method of Bersuder et al. (1998) [44], with slight modifications from Muñoz-Rosique et al. (2023) [45]. A standard was initially established using an analog of vitamin E, the reagent TROLOX (6-hydroxy-2,5,7,8-tetramethylchroman-2-carboxylic acid), known for its antioxidant properties, which reacts with the DPPH (2,2-diphenyl-1-picrylhydrazyl) radical. A stock solution of TROLOX (2 mM) was prepared by weighing 12.5 mg of TROLOX and diluting it in 25 mL of ethanol. Subsequently, a second 0.1 mM stock solution of 10 mL was derived from the initial 2 mM stock solution. This subsequent stock solution was prepared by combining 0.5 mL of the first stock solution with 9.5 mL of ethanol. The TROLOX concentrations for the calibration curve were set at 5, 10, 15, 20, 40, and 50 µM. Two milliliters of each concentration were then prepared from the second 0.1 mM stock solution to establish the calibration curve.

The antioxidant activity of the samples was evaluated using a 0.02% (*w*/*v*) solution of the DPPH radical dissolved in ethanol. A mixture consisting of 500 µL of ethanol, 500 µL of non-protein nitrogen fraction of the sample, and 125 µL of the 0.02% (*w*/*v*) DPPH solution was prepared in Eppendorf tubes. For the blank, 500 µL of the sample was replaced with water. Subsequently, the samples were incubated in the dark at room temperature for 1 h, centrifuged at 10,000× *g* for 2 min, and their absorbance was measured at 517 nm, obtaining % DPPH radical scavenging activity.

To represent the antioxidant capacity of the samples, the IC_50_ was determined, and results were expressed as µg/mL ± standard error of the mean. The analyses were performed three times.

### 2.4. Sensorial Profile

The consumer’s panel was 30 nontrained workers (11 men and 19 women aged 25 to 55 years) of the Universidad Católica San Antonio (Murcia, Spain). 

Samples for sensory analysis were meticulously prepared in a differentiated area, following UNE-EN ISO 8589:2010 [46] standards. Previously, the samples were heated until reaching an internal temperature of 65 °C and measured with a ThermoJack thermometer 5020-0553-30.1047 (Dostmann electronic GmbH, Wertheim, Germany). They were then presented on glasses numbered with three-digit random codes and served.

Sensory analysis was determined through a consumer panel with a 5-point hedonic scale, where 1 was “very unpleasant” and 5 was “very pleasant” following ISO 4121:2003 [47] and ISO 6658:2005 [48] suggestions. General appearance, color, aroma, taste, texture, and overall acceptability were the parameters evaluated.

### 2.5. Nutrition and Health Claims on Food: Legal Framework

A common regulation exists for foodstuffs in the mandatory aspects of labeling contained in Regulation (EU) 1169/2011 [49]. However, there are voluntary claims such as nutrition and health claims. Nutrition claims state or suggest that a food has specific beneficial properties. Health claims state or suggest that the nutrition claim has an effect on health. 

For making nutrition and health claims, the legislations contained in Regulation 1924/2006 [34] and Regulation 432/2012 [33], respectively, were consulted.

### 2.6. Statistical Analyses 

All experiments were conducted in triplicate, and the results were expressed with the mean and standard error of the mean (SEM). All statistical analyses of different parameters were computed using Statistica version 10.0 software package (StatSoft Inc., Tulsa, OK, USA). To assess differences between groups, one-way analysis of variance (ANOVA) was applied. Tukey’s LSD test (*p* < 0.05) was performed to determine significant differences between groups. Differences were considered statistically significant when *p* values were equal to or less than 0.05. Relationships among studied factors were presented using appropriate figures and tables. 

## 3. Results and Discussion

### 3.1. Physicochemical and Nutritional Properties

In comparison to the control group (CTRL), algae–vegetable cream (CV-AN) showed significantly higher values (*p* < 0.05) for moisture, protein, amino acid nitrogen (AAN), non-protein nitrogen (NPN) fractions, ash, fat, and fiber, as depicted in Table 1. In contrast, there were no significant differences (*p* > 0.05) observed for the other remaining components, including pH, a_w_, and salt content, between CV-AN and CTRL. In this aspect, Boukid et al. (2021) observed the same pattern of behavior for moisture, fat, protein, and fiber content in a similar vegetable cream [50]. NPN and AAN contents were significantly higher (*p* < 0.05) in CV-AN because of the increment of total protein.

The improvement in the nutritional profile of the algae–vegetable cream (CV-AN) is evident thanks to the protein content provided by golden *Chlorella vulgaris* (CvG) and the iodine content provided by both *Ascophyllum nodosum*, the natural (An) and the smoked one (AnS) (see Section 3.2).

Color characteristics of the CV-AN were significantly affected by the algae addition (*p* < 0.05) in a* and b* chromatic coordinates, while brightness (L*) did not change significantly (*p* = 0.089), as shown in Table 2.

CV-AN resulted in an increment of a*, which made it redder in color, and a decrease in b*, which made it less yellowish compared to CTRL, which was expected by the addition of the brownish-colored An and AnS.

The ΔE* between CTRL and CV-AN was 1.74 and, as defined by the optimum values specified in ISO 12647-2:2016 [51], it is standardized that the human eye does not detect a difference with a ΔE* between one and two. 

Color similarity of CTRL and CV-AN maintains the quality perception of the original product and promotes higher consumer acceptance by eliminating any perceptible disparity that may influence the perception of taste or overall quality.

### 3.2. Bioactivity

Peptides also demonstrate other beneficial functional properties, such as antioxidant and antihypertensive properties, in addition to their nutritional profile. Microalgae peptides stand out as promising angiotensin I-converting enzyme (ACE-I) inhibitors. This is because a growing number of peptides exhibit hypotensive effects and activity similar to ACE-I inhibitors. The antihypertensive activity of Cv has been demonstrated by several authors previously [52,53,54]. 

Brown macroalgae, on the other hand, also possess bioactive compounds with the ability to inhibit ACE-I that are beneficial in the treatment of hypertension, blocking the conversion of angiotensin-I to angiotensin-II, a vasoconstrictor associated with several cardiovascular and renal diseases, such as arterial hypertension and cardiac and renal failure, among others [55]. The fact that brown macroalgae behave as antihypertensive agents has been previously reported by other authors [56,57].

**Figure 1:**
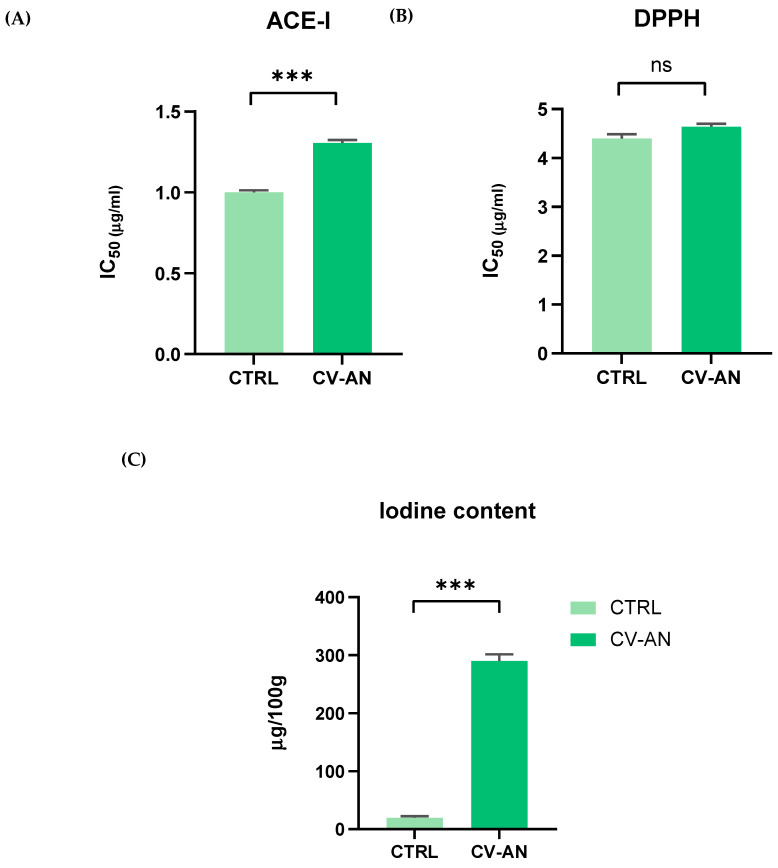
Bioactive activity of vegetable cream (CTRL) versus algae–vegetable cream (CV-AN) represented by antihypertensive activity (**A**), antioxidant activity (**B**), and iodine content (**C**). *p* values were derived from ANOVA; ***: *p* ≤ 0.001; and ns: not statistically significant (*p* > 0.05). Data are mean ± standard error of mean (SEM).

Values represented in Figure 1A shows the peptide concentration required to inhibit 50% of ACE-I activity. It was observed that there were significant differences (*p* < 0.05) between CTRL and CV-AN, revealing that a higher amount of CV-AN was necessary to obtain the same antihypertensive activity as CTRL. The mean and SEM values obtained for CTRL were 1.00 ± 0.01 versus 1.31 ± 0.02 for CV-AN. Future studies are needed to further investigate these results.

Antioxidant capacity was measured by the DPPH method. In this study, no significantly higher values (*p* > 0.05) were found in the antioxidant capacity of CV-AN, indicating that CTRL itself has free radical scavenging capacity, as shown in Figure 1B. 

Brown algae are rich in antioxidant polyphenols, more specifically phlorotannins, which are unique to brown algae. In addition to a demonstrated high antioxidant capacity [17,18,19,58], phlorotannins have a number of promising biofunctional attributes, with beneficial health effects such as anticancer, immunomodulatory, anti-inflammatory, anti-diabetic, and ACE-I inhibitory activities, among others [59]. 

On the other hand, the antioxidant activity is attributed to the presence of various chemical compounds, including chlorophyll derivatives, terpenoids, and carotenoids. Previous studies with Cv have demonstrated the antioxidant effects of the microalgae [60,61] and those included in foods [62,63], however, CvG, having lower chlorophyll content due to a mutation, could decrease the antioxidant effect of CV-AN provided by macroalgae with phlorotannin [17,18]. 

Few studies have been found that include algae in vegetable formulas. In the study carried out by Lafarga et al. [64], where the effect of the incorporation of microalgae in a vegetable soup was analyzed, the antioxidant capacity was studied with the DPPH and FRAP methods, the latter being the one that obtained higher results, exhibiting greater sensitivity in the detection of antioxidant capacity. This analysis could be interesting to better explain this aspect in future studies.

Insufficient iodine intake is related to inadequate thyroid hormone production and may cause mental impairment and thyroid diseases [65]. The World Health Organization (WHO) recommends an iodine intake of 150 μg/day for healthy adults [66], and the tolerable upper intake level (UL) set by EFSA is 600 μg/day in healthy adults [67]. Iodization of table salt is the way most countries comply with the minimum iodine intake. This is especially important for at-risk groups, such as pregnant women, newborns, women of childbearing age, and vegans, who do not consume iodine-containing foods in their regular diet. The content of iodine in brown algae is typically higher than the red or green varieties [68,69]. 

Iodine content, shown in Figure 1C, was obtained in CV-AN and compared to CTRL, the first one resulting in a statistically significant increase (*p* < 0.05) with respect to the second one, obtaining mean values of 290 µg/100 g and 20 µg/100 g, respectively. These values provide evidence that CV-AN could have functional properties related to the proper functioning of the thyroid gland and normal growth, among other health claims.

### 3.3. Sensorial Profile

**Figure 2:**
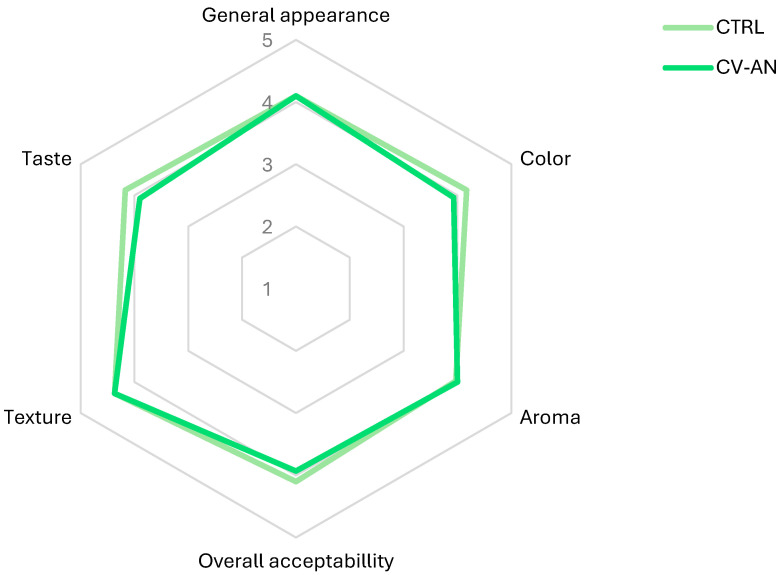
Average sensory panel responses (*n* = 30) to vegetable cream (CTRL) and algae–vegetable cream (CV-AN): 1: very unpleasant; 2: unpleasant; 3: indifferent; 4: pleasant; and 5: very pleasant.

The sensory attributes including general appearance, color, aroma, taste, texture, and overall acceptability were assessed for both CTRL and CV-AN. The findings are depicted in Figure 2, representing the average scores assigned by the consumers for the evaluated parameters.

No significant differences (*p* > 0.05) were observed between CTRL and CV-AN for any of the parameters evaluated. The mean overall acceptability score was quite similar: 4.10 ± 0.13 for CTRL versus 3.93 ± 0.18 for CV-AN. Comments collected from the consumer panel highlighted the lingering smoky taste residue as a positive aspect.

However, the neutral sensorial profile of the CvG used in our study did not worsen the sensory attributes of vegetable cream, unlike others where low acceptability scores were observed, especially at high algae concentrations [64]; therefore, taste is a limitation of this type of marine ingredient [31,34]. In fact, its combined use with An and AnS slightly improved the flavor by imparting smoky notes, although this improvement was not statistically significant.

Due to the similarity, in terms of the overall score of both creams, it can be concluded that the sensory value of CTRL was maintained, adding the organoleptic characteristics of the smoked flavor in addition to the lack of the marine flavor in CV-AN, which is so pronounced in algae. Other authors who incorporated *Chlorella* sp. into foods, even at lower concentrations than in CV-AN, had a negative effect on taste that was reflected in the sensory panel [50,64]. 

### 3.4. Nutrition and Health Claims

#### 3.4.1. Protein Values

In accordance with current nutrition claims legislation [34], to declare CV-AN as a “source of protein” on the label, the protein value must be at least 12% of the total energy value of the vegetable cream. In this case, the protein content of CV-AN accounts for 12.22% of the total energy content of the vegetable cream, compared to 5.9% for CTRL. 

As a result of the food being considered a “source of protein”, it is possible to declare HC appended in Regulation (EU) 432/2012 [33]. The health claims that CV-AN complied with were “protein contributes to a growth in muscle mass”, “protein contributes to the maintenance of muscle mass”, and “protein contributes to the maintenance of normal bones”.

#### 3.4.2. Iodine Values

According to Regulation (EU) 1924/2006 [34], in order to make the nutrition declaration “source of” for vitamins and minerals, it is necessary that the food contains a significant amount. Regulation (EU) 1169/2011 [49] determines that, in foods other than beverages, 15% of the Reference Daily Intake (NRV) can be considered a “significant amount”. In the case of iodine (NRV 150 µg/day), an intake of 22.5 µg of iodine per 100 g of food is considered sufficient to make the nutrition claim “source of iodine”.

The iodine content of CV-AN was 290 µg/100 g, which is 193.3% of NRV, unlike CTRL, which contained 20 µg/100 g, making it unable to be significant. Thus, CV-AN was considered an “iodine source” and, in addition, “high iodine content” for exceeding twice the value of “iodine source” [49]. With an NRV of 150 µg/day of iodine, consumption of 51.72 g/day of CV-AN would be adequate to meet the recommended daily intake of iodine.

The health claims supporting these nutrition claims were “iodine contributes to normal cognitive function”, “iodine contributes to normal energy-yielding metabolism”, “iodine contributes to normal functioning of the nervous system”, “iodine contributes to the maintenance of normal skin”, and “iodine contributes to the normal production of thyroid hormones and normal thyroid function”.

## 4. Conclusions

According to the results obtained, the incorporation of algae in the formulation induced significant changes compared to control vegetable cream (CTRL), as the analysis revealed significant variations in several nutritional components between both vegetable creams. Moisture, ash, protein, fat, iodine, and fiber content showed significant differences indicating the influence of algae incorporation on these parameters. Interestingly, salt concentration remained consistent with no statistical differences, suggesting that the addition of algae did not affect this aspect of the product’s composition. While there were color modifications in algae–vegetable cream (CV-AN), these changes were imperceptible to the human visual perception. This conclusion was further supported by sensory panel responses, of which the results showed no significant differences between CTRL and CV-AN. 

For functional properties, antioxidant activity showed no significant differences between the control sample and algae–vegetable cream, unlike antihypertensive, which experienced an increase in the amount of peptides needed to inhibit 50% of ACE-I activity in CV-AN: 1.31 ± 0.02 versus 1.00 ± 0.01 in CTRL.

A particularly remarkable finding was the statistically higher iodine content in CV-AN, attributed to the presence of both macroalgae. This higher iodine content conferred functional properties in the labeling related to the maintenance of cognitive, nervous, thyroid, and metabolic functions, indicating possible health benefits associated with consumption of CV-AN.

The incorporation of golden *Chlorella vulgaris* (CvG) in a vegetable cream resulted in an innovative food source of protein while *Ascophyllum nodosum* (An) and smoked *Ascophyllum nodosum* (AnS) presented with high iodine contents. In contrast to other studies, sensory evaluations showed no differences between CV-AN and CTRL. Consumers did not detect the typical marine notes of seaweed, suggesting a favorable acceptance. These findings could create commercial opportunities to incorporate this algal blend into a wider variety of food products to enrich them, promoting both ingredient diversity and health benefits. Further research is warranted.

## Figures and Tables

**Table 1:** Physicochemical composition of vegetable cream (CTRL) and algae–vegetable cream (CV-AN).

Physicochemical Properties	CTRL	CV-AN	*p* Value
pH	5.60 ± 0.04	5.68 ± 0.01	0.146
Water activity (a_w_)	0.97 ± 0.0003	0.97 ± 0.0003	0.101
Moisture (%)	90.21 ± 0.16	88.24 ± 0.07	0.000
Ash (%)	0.60 ± 0.04	0.93 ± 0.03	0.003
Protein (N × 6.25) (%)	0.80 ± 0.017	1.91 ± 0.006	0.000
NPN (%)	7.00 ± 0.00	9.33 ± 0.00	0.000
AAN (%)	0.05 ± 0.02	0.18 ± 0.00	0.006
Fat (%)	3.69 ± 0.08	4.40 ± 0.09	0.012
Fiber (%)	0.80 ± 0.03	1.47 ± 0.08	0.001
Salt (%)	0.57 ± 0.03	0.53 ± 0.03	0.519
Carbohydrate *	4.01	3.09	
Energy (kcal/100 g) **	54.05	62.54	

Note: *p* values were derived from ANOVA; *p* < 0.05 was indicated by bold. CTRL: vegetable cream; CV-AN: algae–vegetable cream; NPN: non-protein nitrogen; and AAN: amino acid nitrogen. Data are mean ± standard error of mean (SEM). * Carbohydrate values were calculated by differences. ** Energy was calculated according to Annex XIV conversion factors specified in Regulation (EU) 1169/2011.

**Table 2:** Colorimetry parameters of vegetable cream (CTRL) and algae–vegetable cream (CV-AN).

Colorimetry Parameters	CTRL	CV-AN	*p* Value
L*	65.39 ± 0.09	66.31 ± 0.40	0.089
a*	6.27 ± 0.10	6.69 ± 0.10	0.044
b*	41.49 ± 0.38	37.87 ± 0.31	0.002
ΔE*	-	1.74	

Note: *p* values were derived from ANOVA; *p* < 0.05 was indicated by bold. CTRL: vegetable cream; CV-AN: algae–vegetable cream; L*: lightness; a*: green/red; b*: glue/yellow; and ΔE*: Delta E. Data are mean ± standard error of mean (SEM).

## Data Availability

The data presented in this study are available on request from the corresponding author. The data are not publicly available due to privacy restrictions.
